# Neurotransmitter accumulation and Parkinson's disease‐like phenotype caused by anion channelrhodopsin opto‐controlled astrocytic mitochondrial depolarization in substantia nigra pars compacta

**DOI:** 10.1002/mco2.568

**Published:** 2024-05-15

**Authors:** Sen‐Miao Li, Dian‐Dian Wang, Dan‐Hua Liu, Xiao‐Yan Meng, Zhizhong Wang, Xitong Guo, Qian Liu, Pei‐Pei Liu, Shu‐Ang Li, Songwei Wang, Run‐Zhou Yang, Yuming Xu, Longde Wang, Jian‐Sheng Kang

**Affiliations:** ^1^ Clinical Systems Biology Laboratories The First Affiliated Hospital of Zhengzhou University Zhengzhou China; ^2^ Department of Neurology The First Affiliated Hospital of Zhengzhou University Zhengzhou China; ^3^ The Academy of Medical Sciences Zhengzhou University Zhengzhou China; ^4^ College of Electrical and Information Engineering Zhengzhou University Zhengzhou China; ^5^ Zhengzhou University of Technology Zhengzhou China; ^6^ North China University of Water Resources and Electric Power Zhengzhou China; ^7^ NHC Key Laboratory of Prevention and Treatment of Cerebrovascular Disease Zhengzhou University Zhengzhou China; ^8^ Henan Key Laboratory of Cerebrovascular Diseases Zhengzhou University Zhengzhou China

**Keywords:** anion channelrhodopsin, astrocyte, GABA, glutamate, mitochondria, optogenetics, Parkinson's Disease

## Abstract

Parkinson's disease (PD) is a mitochondria‐related neurodegenerative disease characterized by locomotor deficits and loss of dopaminergic (DA) neurons in the *substantia nigra pars compacta* (SNc). Majority of PD research primarily focused on neuronal dysfunction, while the roles of astrocytes and their mitochondria remain largely unexplored. To bridge the gap and investigate the roles of astrocytic mitochondria in PD progression, we constructed a specialized optogenetic tool, mitochondrial‐targeted anion channelrhodopsin, to manipulate mitochondrial membrane potential in astrocytes. Utilizing this tool, the depolarization of astrocytic mitochondria within the SNc in vivo led to the accumulation of γ‐aminobutyric acid (GABA) and glutamate in SNc, subsequently resulting in excitatory/inhibitory imbalance and locomotor deficits. Consequently, in vivo calcium imaging and interventions of neurotransmitter antagonists demonstrated that GABA accumulation mediated movement deficits of mice. Furthermore, 1 h/day intermittent astrocytic mitochondrial depolarization for 2 weeks triggered spontaneous locomotor dysfunction, α‐synuclein aggregation, and the loss of DA neurons, suggesting that astrocytic mitochondrial depolarization was sufficient to induce a PD‐like phenotype. In summary, our findings suggest the maintenance of proper astrocytic mitochondrial function and the reinstatement of a balanced neurotransmitter profile may provide a new angle for mitigating neuronal dysfunction during the initial phases of PD.

## Graphical abstract

The depolarization of astrocytic mitochondria within the SNc in vivo led to the accumulation of γ‐aminobutyric acid (GABA) and glutamate in SNc, subsequently resulting in excitatory/inhibitory imbalance and locomotor deficits. In vivo calcium imaging and interventions of neurotransmitter antagonists demonstrated that GABA accumulation mediated movement deficits of mice. One hour/day intermittent astrocytic mitochondrial depolarization for 2 weeks triggered spontaneous locomotor dysfunction and the loss of DA neurons, suggesting that astrocytic mitochondrial depolarization was sufficient to induce a PD‐like phenotype.

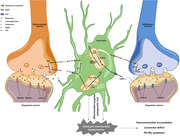

## 1 INTRODUCTION

Parkinson's disease (PD) is a progressive neurodegenerative disease with a common characterization of specific degeneration of dopaminergic (DA) neurons in the *substantia nigra pars compacta* (SNc). These neurons play a critical role in modulating the basal ganglia circuitry, a neural circuit essential for the regulation of both gross and fine motor functions.^1^ The loss of DA neurons in the SNc leads to a reduction in striatal dopamine levels, giving rise to motor symptoms such as resting tremor, bradykinesia, and hypokinesia.^2^ Despite substantial research, a comprehensive understanding of the etiologic mechanism of PD remains challenging. Moreover, the predominant focus of PD research has traditionally centered on neuronal mechanisms, potentially causing a relative neglect of investigations into astrocytic functions. In recent years, mounting evidence has shed light on the multifaceted roles of astrocytes in supporting central nervous system (CNS) homeostasis,^3^, ^4^, ^5^, ^6^, ^7^ including lactate supply,^8^ neurotransmitter metabolism,^9^ the supply of functional mitochondria to neurons^10^ and dysfunctional mitochondria clearance.^11^ Disturbances in normal functions of astrocytes can result in the disruption of homeostasis, giving rise to neurotoxic effects and potentially contributing to neuronal degeneration.^12^, ^13^, ^14^, ^15^, ^16^, ^17^, ^18^ Clearly, recent progresses highlighted the need for in‐depth exploration of astrocytic roles in neurodegenerative diseases, such as PD.

The mitochondrion is a vital eukaryotic organelle involved in multiple physiological processes, including energy generation, heat production and cellular survival.^19^ Increasing evidence has demonstrated their roles in the development of PD.^20^ A recent study has demonstrated that the disruption of neuronal mitochondrial complex I is sufficient to induce a PD‐like phenotype.^21^ On the other hand, mitochondrial functions of astrocytes are also essential for their various physiological roles in the CNS, such as the neurotransmitter metabolisms of glutamate and γ‐aminobutyric acid (GABA).^9^, ^12^, ^13^, ^22^ Several key enzymes involved in neurotransmitter metabolism are located within or associated with mitochondria, including monoamine oxidase B (MAOB),^12^ GABA transaminase (ABAT), and glutamate dehydrogenase (GDH).^9^ As a result, disruptions in astrocytic mitochondrial function could make neurons more vulnerable to dysfunctional astrocytes, potentially leading to progressive neurodegeneration. However, the exact causal roles of astrocytic mitochondria in the progression of PD remain poorly understood due to a lack of suitable research tools.

Optogenetics is an emerging technology that employs genetically encoded, light‐sensitive rhodopsin proteins to selectively manipulate neuronal activities and animal behavior.^23^ Here we reported a mitochondrial‐targetable anion channelrhodopsin by fusing *Guillardia theta* anion channelrhodopsin 1 (GtACR1)^24^ with a four times repeated sequence of cytochrome oxidase 8 (4cox8). Mitochondrial‐targeted GtACR1 remains chloride ion (Cl^−^) conductivity and depolarizes mitochondrial membrane potential (MMP) of neurons and astrocytes in vitro upon light stimulation. By employing mitochondrial‐targeted GtACR1 to induce astrocytic MMP depolarization upon light stimulation within SNc in mice, we observed motor deficits and alterations in neurotransmitter levels. Furthermore, long term 1 h/day intermittent astrocytic mitochondrial depolarization using mitochondrial‐targeted GtACR1 photo‐activation led to a PD‐like phenotype, which was characterized by both locomotor impairments and histopathological changes.

Our results suggest that astrocytic mitochondrial dysfunction is adequate to cause disturbances in neurotransmitter levels and excitatory/inhibitory balance, and elicit a PD‐like phenotype. The maintenance of proper mitochondrial function and the reinstatement of a balanced neurotransmitter profile may provide a new angle for mitigating neuronal dysfunction during the initial phases of PD, thereby paving a way for novel therapeutic strategies.

## 2 RESULTS

### 2.1 Mitochondrial targeting of anion channelrhodopsin GtACR1

To reduce MMP in astrocytes, three strategies were evaluated: (1) Expressing cation‐selective channelrhodopsin to allow cation influx into mitochondria and dissipate MMP; (2) expressing anion‐selective channelrhodopsin to allow anion redistribution according to its equilibrium potential and reduce MMP; (3) targeting an inward proton pump to inner mitochondrial membrane to pump protons into the mitochondrial matrix, collapsing MMP. Considering the effects of cations and proton on mitochondrial metabolism and other off‐target effects,^25^ cation channelrhodopsin and inward proton pump might not be good candidates for reversible MMP depolarization. Whereas, anion‐selective channelrhodopsin could dissipate MMP by altering mitochondrial electrochemical gradient of chloride without causing undesired effects.

**FIGURE 1:**
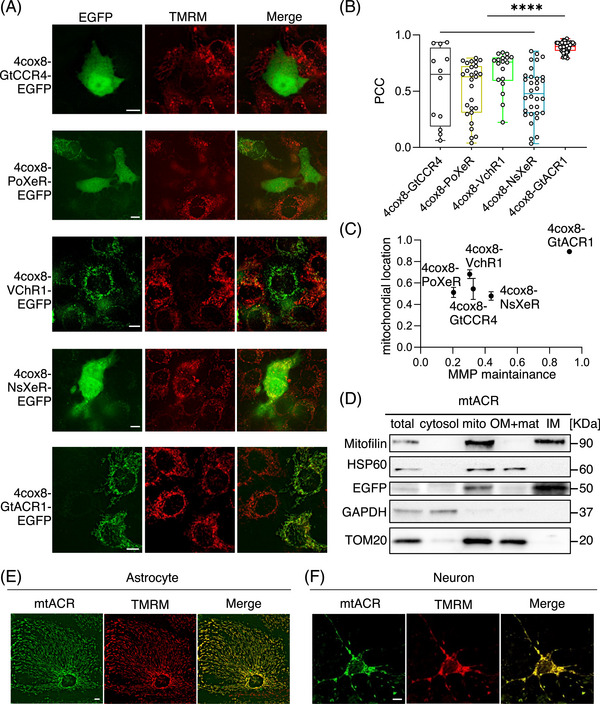
Screening and targeting an anion channelrhodopsin GtACR1 to mitochondria. (A) Representative images of HeLa cells expressing fused channelrhodopsins, which were N‐terminal fused with 4cox8 and C‐terminal fused with EGFP. Scale bars, 10 µm. (B) Quantitative analysis of the colocalization between fused channelrhodopsins and mitochondria indicated by the Pearson Correlation Coefficient (4cox8‐GtCCR4, *n* = 12; 4cox8‐PoXeR, *n* = 27; 4cox8‐VchR1, *n* = 18; 4cox8‐NsXeR, *n* = 32; 4cox8‐GtACR1, *n* = 50; ****p < 0.0001). (C) Mitochondrial location and the capability of fused channelrhodopsins maintaining MMP without photo‐stimulation. (D) The submitochondrial fraction location of mtACR. Mitofilin was used as a marker of mitochondrial inner membrane; EGFP as a marker of fused channelrhodopsin mtACR; HSP60 as the marker of mitochondrial matrix; TOM20 as a marker of mitochondrial outer membrane; GAPDH as the marker of cytosol. (E and F) Representative images of astrocyte and neuron expressing EGFP‐fused mtACR. TMRM was used as the marker of mitochondria. Scale bars, 10 µm.

Consequently, channelrhodopsins such as Volvox channelrhodopsin‐1 (VChR1)^26^ and Guillardia theta cation channelrhodopsin‐4 (GtCCR4),^27^, ^28^ anion channelrhodopsins like GtACR1,^24^ and inward proton pumps such as Parvularcula oceani xenorhodopsin (PoXeR),^29^ Nanosalina xenorhodopsin (NsXeR)^30^ were used for mitochondrial targeting. The efficiency of the mitochondrial targeting was evaluated using Pearson's correlation coefficient (PCC) with a mitochondrial marker, tetramethylrhodamine methyl ester (TMRM). Moreover, staining with TMRM, which is voltage sensitive, was also used to indicate whether these mitochondrial‐targeted microbial rhodopsins could preserve MMP without light illumination. By fusing various mitochondrial signal peptides to microbial rhodopsins, such as ATP Binding Cassette Subfamily B Member 10 (ABCB10) signal peptide (Figure S1A) and 4cox8 (Figures 1A and S1B), we observed that all ABCB10 signal peptide‐fused microbial rhodopsins demonstrated limited capacity of targeting mitochondria (Figure S1A). Meanwhile, 4cox8‐fused GtCCR4, PoXeR, and NsXeR exhibited limited efficacy in mitochondrial targeting, as evidenced by PCC values (in mean ± s.d., standard deviation) of 0.5447 ± 0.3405, 0.5120 ± 0.2464, and 0.4781 ± 0.2241, respectively (Figures 1A–C). VChR1 had a partial mitochondrial targeting capability and a PCC value of 0.6821 ± 0.1745. However, most 4cox8‐VChR1‐transfected cells lost their MMP under unilluminated conditions, which might elicit potential nonspecific effects.

Among these signal peptide‐fused microbial rhodopsins, 4cox8‐GtACR1 exhibited optimal mitochondrial targeting efficiency with a PCC of 0.8920 ± 0.04565 and MMP preservation under normal conditions (Figures 1A–C). Furthermore, western blot of submitochondrial fraction in 4cox8‐GtACR1 stable transfected Hela cells revealed that it exclusively located in inner mitochondrial membrane without leaky expression on outer mitochondrial membrane (Figure 1D). Considering Ca^2+^ and H^+^ are actively involved in mitochondrial metabolism beyond their potential roles in MMP, anion channelrhodopsin is a better and desired candidate for a safe control of MMP. Therefore, we named the mitochondrial‐targeted anion channelrhodopsin GtACR1 as mtACR and used for further applications. We subsequently transduced mtACR into neurons and astrocytes and evaluated their mitochondrial targeting. Our results confirmed the mitochondrial localization and preservation of MMP of mtACR expressed on neurons and astrocytes (data in mean ± s.d.: for neurons, PCC = 0.88 ± 0.07551, *n* = 17; for astrocytes, PCC = 0.86 ± 0.06461, *n* = 36) (Figures 1E, F and S1C).

### 2.2 Photo‐stimulation of mtACR induces mitochondrial depolarization

GtACR1 exhibits anion ion permeability with following tendency: NO_3_^−^ > I^−^ > Br^−^ > Cl^−^ > F^−^ > SO_4_ ^2^ ^−^ = Asp^−^.^24^ As Cl^−^ is the major physiological anion, GtACR1 primarily acts as a Cl^−^ channel in vivo. GtACR1 exhibited varying permeability under various wavelengths, so that we characterized its action spectrum via the whole‐cell patch‐clamp of HEK293t cells. GtACR1 elicited a peak photocurrent at 500 ± 20 nm (Figure S2A), so subsequent photo‐stimulation was performed with blue‐green light. To verify that GtACR1 possesses Cl^−^ conductivity, we cotransfected cultured astrocytes with GtACR1 and a ratiometric Cl^−^ indicator, clomeleon.^31^ Clomeleon consists of two fluorescence proteins, CFP (Cyan Fluorescent Protein) and YFP (Yellow Fluorescent Protein), connected by a short linker. As the fluorescence of YFP is sensitive to Cl^−,^ the ratio of YFP/CFP is related to the concentration of Cl^−^. When astrocytes transfected with GtACR1 were subjected to photo‐stimulation, an increase in the fluorescent ratio was observed after light stimulation, indicating a Cl^−^ flowing into the cytosol (Figures S2B–D). The result demonstrated that photo‐stimulation of GtACR1 induced a Cl^−^ photo‐current; in contrast, control cells without GtACR1 incubated in Cl^−^‐free Tyrode's medium exhibited no significant changes in fluorescent ratio, indicated that the increase caused by light in GtACR1‐transfected cells was not an artifact of light stimulation (Figures S2B–D). To further verify GtACR1 activity, we expressed GtACR1 on neuronal plasma membrane (Figure S2E) and test its effect on neuronal firing inhibition (Figure S2F). As expected, light stimulation inhibited action potentials evoked with current injection. Meanwhile, light had no effect on action potentials of mtACR‐transfected neurons, indicating a negligible leakage of mtACR on plasma membrane (Figures S2G–H).

**FIGURE 2:**
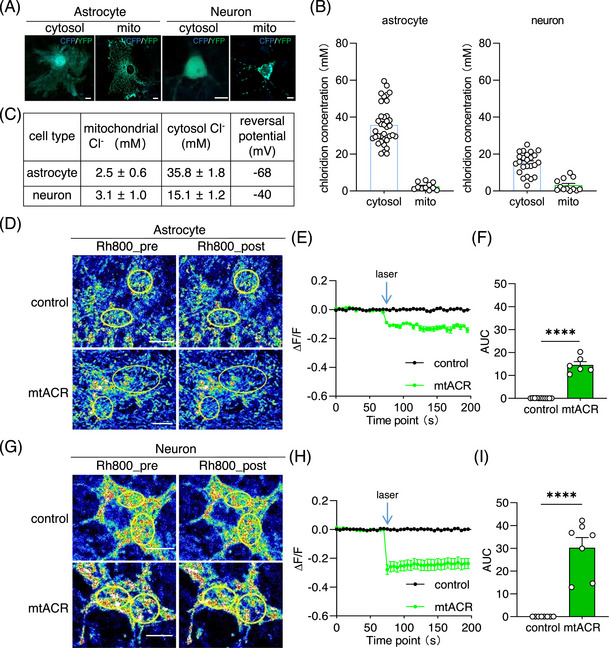
mtACR under photo‐stimulation‐induced mitochondrial depolarization. (A) Representative images of astrocyte and neuron expressing clomeleon or 4cox8‐clomeleon to determine Cl^−^ concentrations of cytosol and mitochondrial. Scale bars, 10 µm. (B) Concentrations of mitochondrial Cl^−^ and cytosol Cl^−^ in neuron and astrocyte measured with clomeleon (astrocytic cytosol, *n* = 36; astrocytic mitochondria, *n* = 11; neuronal cytosol, *n* = 24; neuronal mitochondria, *n* = 12). (C) Summarized concentrations of mitochondrial Cl^−^ and cytosol Cl^−^ in neuron and astrocyte, and the equilibrium potential calculated with Nernst equation. (D, G) Representative images of astrocyte and neuron expressing mtACR or only fluorescent protein, respectively. Rhodamine 800 (Rh800) was used as the indicator of MMP. Left of each panel showed the fluorescence of Rh800 before photo‐stimulation. Right of each panel showed the fluorescence of Rh800 after photo‐stimulation. Scale bars, 10 µm. (E and H) Normalized ratio of Rh800 fluorescent intensity changed with photo‐stimulation. Black represented control group (*n* = 11 for astrocyte; *n* = 8 for neuron), while green points represented mtACR group (*n* = 6 for astrocyte; *n* = 7 for neuron). (F, I) Areas under the curves of fluorescent ratio of Rh800. All data of figures in mean  ±  s.e.m.; ****p < 0.0001.

As a Cl^−^ channel, the membrane potential is shifted towards its equilibrium potential once the channel is opened. The direction of Cl^−^ flow mediated by mtACR on mitochondria depends on the equilibrium potential of chloride across the mitochondria inner membrane. To use the Nernst equation to calculate equilibrium potentials, we utilized the chloride indicator clomeleon to quantify the Cl^−^ concentrations in cytosol of neurons and astrocytes (Figures 2A and B). By targeting clomeleon to mitochondrial matrix using 4cox8, the concentrations of Cl^−^ within mitochondrial matrix were determined (Figures 2A–C). The calculated equilibrium potentials for astrocytes and neurons mitochondria were −68 and −40 mV, respectively (Figure 2C). The results indicated that mtACR photo‐activation could lead to a depolarization of mitochondria, which had a resting inner membrane potential approximately at −100 to −180 mV under physiological conditions.^32^, ^33^ By using cell specific promoter, we expressed mtACR in neurons or astrocytes via adeno‐associated virus (AAV) transduction. MMP was monitored using a MMP‐sensitive fluorescent dye, rhodamine 800.^34^, ^35^ Upon photo‐stimulation of mtACR, we observed significant reductions in fluorescence intensities of rhodamine 800 in both neurons and astrocytes with different amplitudes compared with control group (Figures 2D–I). Notably, the depolarization amplitude in neurons was more prominent than in astrocytes, consistent with the results that neuronal mitochondria possess a higher (less negative) chloride equilibrium potential compared with astrocytic mitochondria (Figure 2C).

Given the pivotal roles of mitochondria in the synthesis of adenosine triphosphate (ATP) and the generation of reactive oxygen species (ROS), we next investigated the effects of activating mtACR on ATP and ROS levels. We cotransfected mtACR‐Mcherry and reconstructed iATP,^36^ a fluorescent sensor of intracellular ATP into primary cultured astrocytes. We found that the photo‐stimulation of mtACR would induce a reduction of fluorescent intensity of iATP, which indicated that the activation of mtACR reduced the production of ATP (Figures S3A–C). As for ROS, we transfected mtACR‐enhanced green fluorescent protein (EGFP) into primary cultured astrocytes. Utilizing the ROS‐sensitive fluorescent dye mitoSOX, we quantified the ROS generation after photo‐stimulation of mtACR on astrocytic mitochondria. We found that the fluorescent intensity of mitoSOX did not change compared with the control group which only expressed EGFP, which indicated that the activation of mtACR did not change the generation of ROS (Figures S3D–F). These findings demonstrated a selective effect of mtACR‐induced MMP depolarization on mitochondrial ATP production but without elevating ROS levels.

### 2.3 Astrocytic mitochondrial depolarization causes locomotor deficit

**FIGURE 3:**
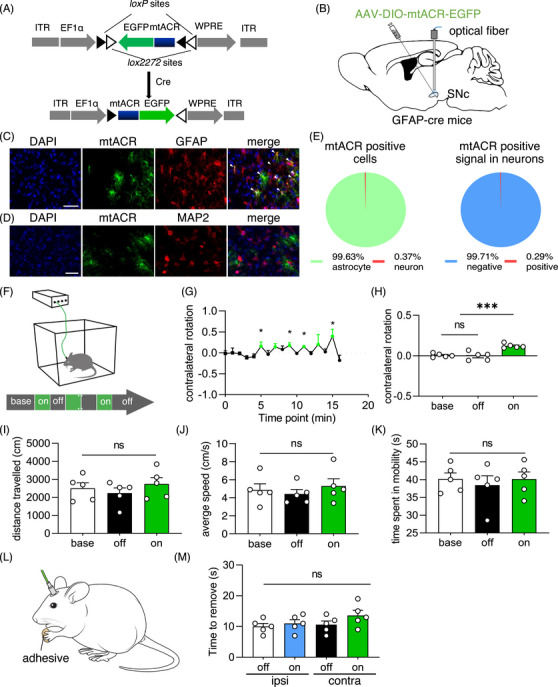
Transient astrocytic mitochondrial depolarization caused reversible locomotor deficit. (A) Virus design and construction of AAV expressing mtACR. (B) Schematic diagram of the virus injection and optical fiber implantation in GFAP‐cre mice. AAV was injected to unilaterally SNc. (C and D) Representative images of GFAP (C) or MAP2 (D) in SNc of GFAP‐cre mice transduced with AAVDJ‐DIO‐mtACR‐EGFP. Scale bars, 100 µm. (E) Left: Positive percentage of mtACR expression in astrocytes (*n* = 543). Right: Negative percentage of mtACR positive signal in neurons (*n* = 690). (F) Schematic diagram of behavior test. (G) Contralateral rotation of time points of behavior tests of mice injected mtACR virus under photo‐stimulation (*n* = 5). (H) Contralateral rotation quantifications of baseline, laser off epochs, laser on epochs of mice received mtACR virus injection (*n* = 5). (I) Distance travelled of each period (*n* = 5). (J) Average speed of mobility of each period (*n* = 5). (K) Time spent in mobility of each period (*n* = 5). (L) Schematic diagram of the adhesive remove test. (M) Time to remove the adhesive of forepaws of mice received mtACR virus (*n* = 5). All data of figures in mean  ±  s.e.m.; *p < 0.05, ***p < 0.001, ns, no significance.

To explore the potential effects of mtACR controlling mitochondrial depolarization, particularly in relation to motor deficits observed in PD, we selectively expressed mtACR in astrocytic mitochondria of SNc using a Cre–loxP system. Glial fibrillary acidic protein (GFAP)‐Cre mice were subjected to unilateral stereotactic injections of AAV expressing mtACR (AAVDJ‐DIO‐mtACR‐EGFP) or a control EGFP marker (AAVDJ‐DIO‐4cox8‐EGFP) in SNc. Subsequently, an optical fiber was implanted over SNc for light delivery (Figures 3A and B). Followed by a 2‐week period for viral expression, animals were placed in an open‐field box for light stimulation and behavior recording. Rotational behavior test, a common method to evaluate gross motor function in unilateral PD models,^37^, ^38^ and adhesive removal test, a behavior test to assess fine motor skills^21^ were utilized to evaluate locomotor function. Afterwards, the mice were euthanized, and brain tissues were harvested for further analysis. Brain slices of these AAV injected mice revealed almost no leakage expression of mtACR in neurons (few colocalization might be due to spatial overlap), as evidenced by staining with GFAP (a marker for astrocytes) and microtubule‐associated protein 2 (MAP2, a neuronal marker) antibodies (Figures 3C–E). The expression of mtACR in astrocytic mitochondria was also confirmed by using the primary‐cultured hippocampal neuron and glia transduced by AAV expressing GFAP‐mtACR. Immunohistochemical analysis was performed using antibodies against GFAP, MAP2, and mitofilin (a mitochondrial marker) (Figures S4A and B). The results confirmed the astrocytic mitochondrial expression of GFAP‐mtACR.

GFAP‐Cre mice injected with AAVDJ‐DIO‐mtACR‐EGFP exhibited contralateral rotation in response to photo‐stimulation, which was reversible upon the cessation of laser exposures (Figures 3F–H). In contrast, mice administered with a control virus did not exhibit locomotor change (Figure S5A). For the reversible locomotor phenotype induced with mtACR photo‐activation, there were no discernible differences in the traveled distance, average speed, or time spent in mobility upon photo‐stimulation compared with baseline or laser‐off period (Figures 3I–K). In addition, the time required for mice to remove adhesive from their contralateral forepaw, relative to their ipsilateral forepaw, also did not show alteration (Figures 3L and M ). To eliminate concerns of potential mtACR leakage onto the astrocytic plasma membrane, we employed AAVDJ‐DIO‐ACR‐EGFP to express GtACR1 specifically to astrocytic plasma membrane in the SNc of GFAP‐Cre mice. Photo‐stimulation did not yield significant locomotor alterations, completely ruling out any potential mtACR leakage as the cause of observed behavioral changes (Figure S5B). Collectively, these findings suggested that transient astrocytic mitochondrial depolarization under mtACR photo‐activation within SNc reversibly altered gross motor functions but did not affect fine motor skills in mice.

Interestingly, we noticed that 6‐hydroxydopamine lesioned animals exhibit ipsilateral rotation following the administration of drugs inducing dopamine release. Drugs activating the dopamine receptor would induce contralateral rotation.^38^ These phenomena indicated that lesion of nigrostriatal pathway would induce the locomotor deficit of ipsilateral side, thus resulting in ipsilateral rotation. However, up to our knowledge, other unilateral lesion forms of SN such as electric damage, injection of GABA, optogenetic inhibition caused contralateral locomotor deficit, thus induced contralateral rotation.^13^, ^39^, ^40^ Clearly, the discrepancies deserve further investigation.

### 2.4 Astrocytic mitochondrial depolarization alters GABA and glutamate levels

To elucidate the mechanisms underlying locomotor deficits induced by astrocytic mitochondrial depolarization by mtACR photo‐activation, we examined the potential effects of mtACR photo‐activation on astrocytes, microglia, and dopamine (DA) neurons. Immunohistochemical analyses revealed no significant alterations in the number of astrocytes or microglia in mtACR‐activated site compared with unstimulated contralateral site, as evidenced by staining with GFAP and ionized calcium‐binding adapter molecule 1 (Iba1) antibody, respectively (Figures S6A–D). In addition, immunostaining for tyrosine hydroxylase (TH), a crucial enzyme for dopamine synthesis as a marker of DA neuron, did not reveal any reduction in its expression in mtACR‐activated site compared with unstimulated contralateral site (Figures S6E–G). Collectively, these findings indicated that the observed locomotor alterations induced by transient astrocytic mitochondrial depolarization under mtACR photo‐activation were not attributed to any histopathological changes of astrocytes, microglia or DA neurons, which were also consistent to the reversible gross motor functions.

**FIGURE 4:**
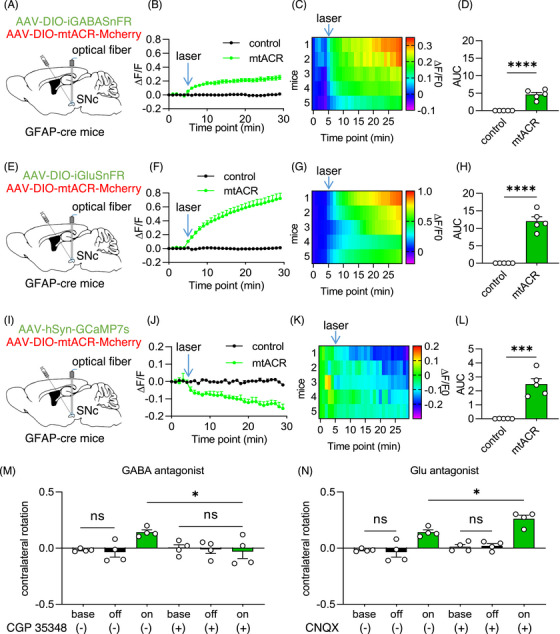
The aberrant levels of GABA and Glutamate caused by astrocytic mitochondrial depolarization. (A, E, and I) Schematic diagram of fiber photometry of iGABASnFR (A), iGluSnFR (E), GCaMP7s (I) coexpressed with mtACR or only fluorescent protein. (B, F, and J) Normalized fluorescent intensity of iGABASnFR (B), iGluSnFR (F), or GCaMP7s (J) coexpressed with mtACR (green) or control virus (black) on astrocytes in SNc under photo‐stimulation (*n* = 5). (C, G, and K) Heat map of GABA (C), glutamate (G), or Ca^2+^ (K) changes (*n* = 5). (D, H, and L) Area under the curve of fluorescent ratio of iGABASnFR (D), iGluSnFR (H) or GCaMP7s (L) (*n* = 5). (M and N) Contralateral rotation quantifications of mice received mtACR virus injection pretreated with saline, CGP35348 (10 mg/kg) or CNQX (1.5 mg/kg) (i.p.) (*n* = 4). All data of figures in mean  ±  s.e.m.; *p < 0.05, ***p < 0.001, ****p < 0.0001, ns, no significance.

Astrocytic mitochondria are known to play critical roles in the neurotransmitter metabolisms of GABA and glutamate,^9^, ^12^, ^13^, ^22^ which are implicated in regulating locomotion^41^ and various neurodegenerative diseases.^12^, ^13^, ^14^, ^15^, ^16^, ^17^, ^18^, ^42^, ^43^, ^44^, ^45^, ^46^ We expected that astrocytic mitochondrial depolarization by mtACR photo‐activation might affect neurotransmitter metabolism, leading to excitatory/inhibitory imbalance and locomotor dysfunction. To test this proposal, we used in vivo photometric techniques to measure glutamate and GABA levels using fluorescent sensors iGABASnFR^47^ and iGluSnFR,^48^ respectively. Interestingly, upon photo‐stimulation, we observed increased concentrations of glutamate and GABA in mice injected with mtACR virus compared with control group that only expressed fluorescent protein (Figures 4A–H). To be cautious, considering these fluorescent sensors were qualitative not quantitative probes, the relative amplitudes of fluorescent intensity might be related to different affinities or sensitivities of probes with neurotransmitters rather than neurotransmitter concentrations.

To test the potential consequence of altered excitatory/inhibitory imbalance, we employed GCaMP7s, a Ca^2+^ fluorescent sensor, to measure neuronal activity upon mtACR activation in vivo. The results demonstrated a decrease in GCaMP7s fluorescence in SNc upon mtACR activation (Figures 4I–L), suggesting the accumulation of GABA played a dominated role in excitatory/inhibitory imbalance caused by mtACR photo‐activation in SNc. To further confirm the alteration of neuronal activity, we applied the voltage indicator ASAP3^49^ to record the voltage alteration of single neuron of brain slices. The result showed that the firing rate of neuron of mtACR group decreased by photo‐stimulation, while control group showed no significant change (Figures S7A–C). These data further confirmed that GABA played a dominated role in excitatory/inhibitory imbalance. Consistently, western blotting showed that mitochondrial anchored enzyme ABAT significantly decreased following photo‐stimulation (Figures S8A and B). The expression of GDH showed decrease (Figure S8C), but there was no statistical significance (Figure S8D). These data suggested that astrocytic mitochondrial depolarization might cause the decrease of the mitochondrial enzymes related to neurotransmitter catabolism, and induce an accumulation of neurotransmitters.

Considering both GABA and glutamate increased during mtACR activation, we further injected blood‐brain barrier permeable GABA receptor antagonist CGP35348 and glutamate receptor antagonist CNQX in mice intraperitoneally before mtACR activation, respectively. Consistent to the results of in vivo calcium imaging, CGP35348 effectively improved motor impairments induced by mtACR activation (Figure 4M, control results in Figure S9A). In contrast, CNQX exacerbated these deficits (Figure 4N, control results in Figure S9B), which consistently supported that the abnormal level of GABA was the main factor for motor dysfunction caused by astrocytic mitochondria depolarization under mtACR photo‐activation. To exclude potential nonspecific effects of general activity change, we compared the distance travelled of mice received antagonists or saline and demonstrated that the nonspecific effects could be excluded (Figures S9C and D).

### 2.5 Daily intermittent astrocytic mitochondrial depolarization induces PD‐like phenotype

Previous studies implicated elevated levels of GABA^12^, ^13^ and glutamate^15^, ^16^, ^17^, ^18^, ^42^, ^43^, ^44^, ^45^, ^46^ as contributing factors to the pathophysiology of PD. Our current findings demonstrated that transient astrocytic mitochondrial depolarization altered neurotransmitter metabolism and led to reversible locomotor deficits. However, PD is a chronic neurodegenerative disorder, suggesting that negative factors underlying the disease may persist over time. Therefore, we investigated whether long‐term intermittent astrocytic mitochondrial depolarization could lead to progressive parkinsonian motor symptoms.

**FIGURE 5:**
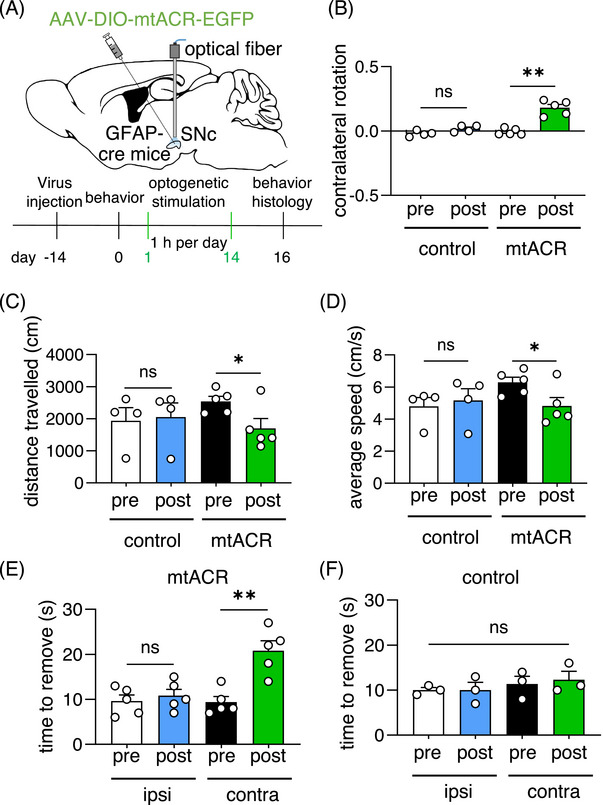
Daily intermittent astrocytic mitochondrial depolarization for 2 weeks induced a PD‐like locomotor deficit. (A) Schematic diagram of 1 h/day intermittent astrocytic mitochondrial depolarization for 2 weeks. (B) Contralateral rotation of mice received daily intermittent optogenetic stimulation (*n* = 5 for mtACR group; *n* = 4 for control group). (C) Distance travelled of pre and post photo‐stimulation of mice injected with mtACR virus or control EGFP virus (*n* = 5 for mtACR group; *n* = 4 for control group). (D) Average speed of pre and post photo‐stimulation of mice injected with mtACR virus or control EGFP virus (*n* = 5 for mtACR group; *n* = 4 for control group). (E and F)The time of mtACR mice (E) or control mice (F) received long‐term optogenetic stimulation to remove the adhesive (*n* = 5 for mtACR group; *n* = 3 for control group).

Mice injected with either mtACR AAV or control AAV (4cox8‐EGFP) in the SNc were subjected to photo‐stimulation for 1 h/day and lasted for 2 weeks. Animal behaviors were recorded before and after the daily intermittent light stimulation protocol (Figure 5A). We observed that mice with mtACR activation exhibited spontaneous contralateral rotation even in the absence of photo‐stimulation after the daily intermittent astrocytic mitochondrial depolarization (Figure 5B). Furthermore, these mice showed significantly decreased traveled distance (Figure 5C) and average speed (Figure 5D) poststimulation. Additionally, the time required to remove adhesive from the contralateral forepaws relative to the ipsilateral forepaws also significantly increased in daily intermittent‐activated mtACR mice (Figure 5E). In contrast, control mice did not display changes (Figure 5F). These data suggested that both gross motor and fine motor function impaired following prolonged intermittent depolarization of astrocytic mitochondria, which was consistent with the motor symptoms of PD.

**FIGURE 6:**
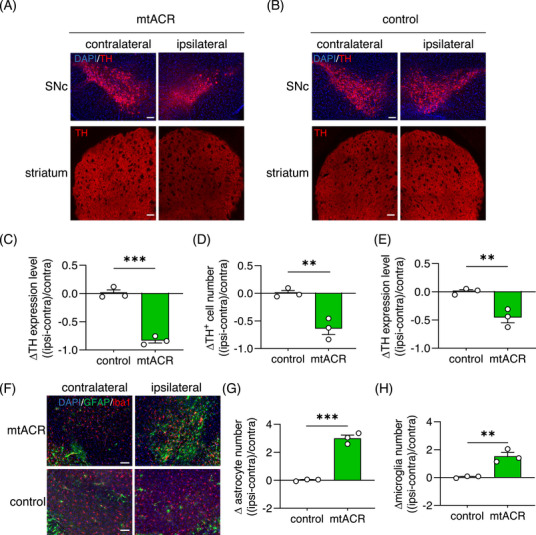
Daily intermittent astrocytic mitochondrial depolarization induced a PD‐like pathological change. (A and B) Representative images of TH in SNc (upper) or striatum (bottom) of mice received mtACR (A) or control virus (B) injection. Scale bars, 100 µm. (C) Relative change of TH expression level in ipsilateral SNc compared with contralateral SNc of mice received mtACR or control virus injection (*n* = 3). (D) Relative change of DA neuron numbers in ipsilateral SNc compared with contralateral SNc of mice received mtACR or control virus injection (*n* = 3). (E) Relative change of TH expression level in ipsilateral striatum compared to contralateral striatum of mice received mtACR or control virus injection (*n* = 3). (F) Representative images of GFAP (green) and Iba1 (red) in SNc of mice received mtACR or control virus injection. Scale bars, 100 µm. (G and H) Relative change of astrocyte (G) and microglia (H) numbers in ipsilateral SNc compared with contralateral SNc of mice received mtACR or control virus injection (*n* = 3). All data of figures in mean  ±  s.e.m.; *p < 0.05, **p < 0.01, ***p < 0.001, ns, no significance.

A common histopathological hallmark of PD is DA neuronal loss. Mice with prolonged intermittent astrocytic mitochondrial depolarization by mtACR photo‐activation were euthanized and brain tissues were harvested for TH staining. We observed significantly decreased TH expressions in ipsilateral SNc and striatum, and TH positive cell number in SNc at mtACR photo‐activated side compared with unstimulated contralateral side (Figures 6A–E). Moreover, considering α‐synuclein (α‐syn) as a crucial hallmark of PD, we performed α‐syn staining in SNc. Strikingly, the result showed that α‐syn aggregated in the ipsilateral side of SNc compared with contralateral side, while the control mice showed no significant change (Figures S10A and B). These results indicated that prolonged astrocytic mitochondrial depolarization sufficiently led to reduction of dopamine synthesis, aggregation of α‐syn and DA neuronal loss. Moreover, we further confirmed that GABA increased in the ipsilateral SN compared with the contralateral side after daily intermittent astrocytic mitochondrial depolarization (Figures S11A and B).

To investigate whether astrocytic mitochondrial dysfunction led to the alteration of astrocytes and microglia, we performed GFAP and Iba1 staining in the SNc. Interestingly, the astrocytic mitochondrial dysfunction did not cause the demise of astrocytes, but increased the number of astrocytes and microglia (Figures 6F–H). The activations of astrocytes and microglia were hallmarks of neurodegenerative diseases.^4^, ^5^, ^6^, ^7^ These findings suggested that 1 h/day intermittent astrocytic mitochondrial depolarization by mtACR photo‐activation for 2 weeks sufficiently resulted in a PD‐like phenotype, evidenced by the locomotor symptoms and pathological changes.

## 3 DISCUSSION

Astrocytes, with their multifaceted functions including maintaining the blood‐brain barrier, regulating synaptic activity, and providing metabolic support to neurons, have emerged as important players in neurodegeneration.^3^ Emerging evidence suggests that astrocytic dysfunction can lead to hypometabolism, ROS generation, and abnormal calcium signaling, contributing to neurodegenerative disease pathogenesis.^3^ As astrocytes are vital for supporting CNS, astrocytic mitochondria play active roles in maintaining normal neuronal functions. Several crucial functions of astrocytic mitochondria in maintaining CNS homoeostasis were well established, especially, astrocytes as energy station such as lactate supply^8^ and functional mitochondrial transferring,^10^ as well as recycling station for neurotransmitter detoxifying and recycling,^9^ and for dysfunctional neuronal mitochondria clearance.^11^ Astrocytic mitochondria also involve in the antioxidative/oxidative and calcium homoeostasis.^22^ In this study, we have developed a GtACR1‐based optogenetic tool mtACR that could manipulate the MMP of astrocytic mitochondria, and demonstrated that daily intermittent astrocytic mitochondrial depolarization for weeks is sufficient to induce the symptom of locomotor deficit, α‐syn aggregation and the loss of DA neurons—key features of PD.

In our screen, three possibilities of controlling MMP were considered to target photo‐activable microbial rhdopsins to mitochondria, including cation channelrhodopsin, anion channelrhodopsin, and inward proton pump. Channelrhodopsin 2 was reported to be targeted to modulate mitochondrial metabolism by fusing with a signal peptide 4cox8.^25^ Here, among five tested microbial rhodopsins (Figure 1), the anion channelrhodopsin GtACR1 was the optimal microbial rhodopsin for mitochondrial targeting with least direct effect on mitochondrial matter metabolism compared with cation or proton. Importantly, the mitochondrial counterpart mtACR of GtACR1 did not affect normal mitochondrial morphology or disrupt membrane potential under unilluminated conditions (Figure 1). In astrocytes, the equilibrium potential of chloride on mitochondrial inner membrane was −68 mV (Figures 2A–C). Therefore, mtACR under photo‐activation functioned as a chloride channel and depolarized astrocytic MMP under physiological conditions, which was confirmed with rhodamine 800 staining assay (Figures 2D–F).

By targeting mtACR to astrocytes in SNc, we observed motor deficits caused by mtACR photo‐activation. An interesting finding was that mtACR activation in astrocytes caused the accumulations of GABA and glutamate, and excitatory/inhibitory imbalance. Astrocytes utilize excitatory amino acid transporters (EAAT) and GABA transporters (GAT) to uptake excess glutamate and GABA from synaptic cleft.^9^ Astrocytic mitochondria contain essential enzymes involved in neurotransmitter metabolism, such as succinic semialdehyde dehydrogenase (SSADH), ABAT, and GDH, which are key enzymes for the catabolism of glutamate and GABA.^9^ The maintenance of MMP is crucial for the translocation of mitochondrial proteins and the transportation of metabolites.^19^, ^32^, ^33^, ^50^ Consistently, astrocytic mitochondrial depolarization caused by mtACR photo‐activation decreased the activities of these enzymes possibly via inhibiting substrate transportation or promoting mitophagy and resulted in the accumulation of GABA and glutamate at synaptic cleft. In vivo calcium imaging and transmitter antagonist interventions demonstrated that it was GABA accumulation that mainly mediated excitatory/inhibitory imbalance and locomotor deficits of mice (Figure 4). Glutamate in SNc was reported to increase locomotion, while GABA inhibited locomotion.^41^ Our findings supported that SNc neurons had a greater susceptibility to GABA metabolism,^51^, ^52^ which might have a potential link with α‐syn accumulation via MAOB and putrescine polyamine pathway.^53^

The most inspiring finding was that transient astrocytic mitochondrial depolarization was sufficient to cause reversible locomotor alterations (Figure 3), suggesting the normal functions and excitatory/inhibitory balance of neurons were tightly coupled to astrocytic functions in SNc. Moreover, we found that mice fasted for 24 h could exacerbate reversible locomotor deficit caused by transient astrocytic mitochondrial depolarization (Figure S12). Intriguingly, the finding might explain potential links between malnutrition and some human behaviors, such as hypoglycemia‐related trembling. Together, our findings suggested that the capacity of neuronal metabolism was quite limited, heavily depended on astrocytic metabolism. Considering age‐related reduction in intestinal,^54^ malnutrition of astrocytes might potentially account for some late‐onset PD like symptoms in clinical.

Moreover, we screened the public datasets and found several scRNA‐seq datasets of PD patients and PD model mice (GSE157783 and GSE187012).^55^, ^56^ We analyzed these datasets and found several mitochondrial‐related differential expressed genes (DEGs) and enriched gene ontology (GO) terms in PD patients and PD model mice (Figure S13, S14 and S15). One interesting phenomenon was that the proportion of both astrocyte and microglia of PD patients increased (10.253% in control to 14.621% in PD patients for astrocytes; 5.418% in control to 15.102% in PD patients for microglia) (Figures S13B and C), which was consistent with the data in Figure 6. Among those DEGs and enriched GO terms related to mitochondria in astrocytes of human and mouse, we found several DEGs enriched to the regulation of MMP (Figures S13E and S14B). Taken together, these data also supported that astrocytic mitochondrial dysfunction might play important roles in PD pathogenesis.

## 4 MATERIALS AND METHODS

### 4.1 Animals

Male mice aged 8−10 weeks were used in the experiments. The allocation of experimental group was randomly. All animal care and experiments were performed in accordance with Institutional Animal Care and Use Committee of Zhengzhou University guidelines. All animal experiments have been approved by the Institutional Animal Care and Use Committee of Zhengzhou University.

### 4.2 Plasmid construction

GtACR1, VChR1, GtCCR4, PoXeR, and NsXeR were synthesized by Genewiz and subsequently fused with enhanced green fluorescent protein (EGFP). The ratiometric Cl^−^ indicator Clomeleon was synthesized by Genewiz. The plasmid tagged with Mcherry was constructed by substituting EGFP with Mcherry. These rhodopsins were cloned into pEGFP‐N1 vectors for expression on the cellular plasma membrane. For mitochondrial localization, the N‐terminal of both rhodopsins and Clomeleon were fused with four repetitive sequences of 4cox8. The plasmids pAAV.GFAP.iGABASnFR (Addgene plasmid # 112172; http://n2t.net/addgene:112172; RRID:Addgene_112172) and pAAV.GFAP.iGluSnFR.WPRE.SV40 (Addgene plasmid # 98930; http://n2t.net/addgene:98930; RRID:Addgene_98930) were generously provided by Loren Looger.

### 4.3 Electrophysiology

All cell patch clamp recordings were performed with a system including Axopatch 700B amplifier (Molecular Devices), Digidata 1440A digitizer (Molecular Devices), and Optoscan monochromator (Cairn Research Ltd.) at room temperature. Micropipettes were pulled by a micropipette puller (Shutter Instruments), positioned by micromanipulator MP‐285 (Shutter Instruments) and filled with intracellular buffer (potassium gluconate 120 mM, NaCl 8 mM, CaCl_2_ 0.5 mM, HEPES 10 mM, ATP‐Mg 2 mM, GTP 0.3 mM, KCl 3 mM, EGTA 5 mM at pH 7.2) filled. The Tyrode's buffer (NaCl 145 mM, KCl 3 mM, HEPES 10 mM, glucose 10 mM, pH 7.4) was used as extracellular buffer. The Cl^−^‐free Tyrode's buffer (sodium gluconate 145 mM, potassium gluconate 3 mM, HEPES 10 mM, glucose 10 mM, pH 7.4) was prepared by replacing Cl^−^ with gluconate ion. Prior to photo‐current recording, all‐trans retinal was added to the cell culture medium 1 h after transient plasmid transfection, and cells were cultured for an additional 12 h. The voltage clamp mode was used for photo‐current recording of HEK 293t cells and measured at 0 mV unless stated otherwise. Cultured neurons were voltage clamped at −70 mV and recorded at current clamp mode at DIV 8−12 after transfection at DIV 4−5. An objective (Olympus) with a 3.315 mW/mm^2^ intensity light generating from a Xenon bulb was applied for the recording of the action spectrum. A monochromator was used to generate 300 ms light pulses with 20 nm bandwidth and various wavelengths ranging from 350 to 750 nm. Imagings were captured with a microscopy (Olympus).

### 4.4 Cell lines and transient expression of combined channelrhodopsins

HEK 293t and HeLa cells were cultured in Dulbecco's modified Eagle medium (Gibco) with 10% fetal calf serum (Gibco) at 37°C, 5% CO_2_. The method of calcium phosphate‐DNA coprecipitation was applied for transient expression in cells.

### 4.5 Stable transfected cell line

Electroporations and lentivirus transductions were used for the construction of stable transfected cell lines. As for electroporation, cells were given electric pulses in an electroporation cup filled with PBS. G418 (100 µg/mL) was applied for stable screening. Single clones with high expression level survived in 0.7 mg/mL Geneticin (Gibco, Life Technologies) were screened with confocal microscope and isolated for further culture. In the case of lentivirus transduction, cells were cultured in a medium containing the virus for 3 days, followed by the application of puromycin (20 µg/mL) for stable screening. After sustained culture, DNA samples of the cell lines were extracted and the existence of plasmid sequence was verified by PCR and sequencing.

### 4.6 Isolation of mitochondria

The stable transfected HeLa cell line of mtACR‐EGFP was digested and collected to the microcentrifuge tube. Then, the pelleted cells were resuspended with 1 mL mitochondrial isolation buffer (250 mM sucrose, 5 mM HEPES, 1 mM EGTA at pH 7.4) and the cell suspension was homogenized with a homogenizer. The operation should be gentle, and the fragmented efficiency should be less than 50%, verified by Trypan Blue staining, to prevent the fragmentation of mitochondria. Following a 15‐s vortex and a subsequent 15‐min settling period, the homogenate was centrifuged at 500×*g*, 4°C for 10 min, and the supernatant was collected. This centrifugation process was reiterated until no precipitates were discernible. The supernatant was then subjected to centrifugation at 10,000×*g*, 4°C for 15 min, yielding isolated mitochondria in the pellet and cytosol in the supernatant. The pellet was resuspended in 1 mL of mitochondrial isolation buffer and centrifuged at 10,000×*g*, 4°C for 10 min, followed by pellet collection. This washing procedure was repeated several times to remove remaining cytosol. The pellet was subsequently collected and suspended with 100 µL mitochondrial isolation buffer and 50 µL lysis buffer (1% Triton X‐100 and 1% Sodium deoxycholate in PBS) to acquire the mitochondria. All of these operations should be bathed with ice.

### 4.7 Isolation of sub‐mitochondrial fractions

The isolated mitochondria were suspended with 100 µL mitochondrial isolation buffer and 50 µL digitonin buffer containing appropriate concentration of digitonin and vibrate it by vortex for 10 min to fragment the mitochondrial outer membrane. Centrifuge the suspension at 10,000×*g*, 4°C for 10 min and collect the supernatant as mitochondrial outer membrane and mitochondrial matrix. Resuspend the pellet with mitochondrial isolation buffer and wash it by centrifugation at 10,000×*g*, 4°C for 10 min. Repeat this for several times and resuspend the pellet with 100 µL membrane extraction buffer A (Beyotime). Centrifuge the suspension at 10,000×*g*, 4°C for 10 min and collect the pellet as mitochondrial inner membrane. Wash it with mitochondrial isolation buffer and add 50 µL membrane extraction buffer B (Beyotime) to dissolve the mitochondrial inner membrane. All these operations should be bathed with ice.

### 4.8 Western blotting

The cytosol, mitochondria, and sub‐mitochondrial fractions were acquired by protocol described above. To get the total cell content, the mtACR‐EGFP stable transfected cells were washed with PBS and homogenized with lysis buffer. For neurotransmitter‐related enzymes, mice received mtACR or control virus injection were sacrificed following 1 h intermittent photo‐stimulation and the brain tissues were collected. Ten microliters of protein sample was loaded onto SDS‐PAGE gel. Proteins were electrophoresed and then transferred to nitrocellulose filter membranes. The membranes were then blocked by quick blocking buffer (Beyotime) for 15 min and incubated overnight at 4°C with the primary antibodies against GFP (ab290; Abcam), mitofilin (10179‐1‐AP, Proteintech), Heat Shock Protein 60 (HSP 60) (ADI‐SPA‐806, Enzo Life Sciences), TOM 20 (11802‐1‐AP, Proteintech), glyceraldehyde‐3‐phosphate dehydrogenase (GAPDH) (ab70699; Abcam), ABAT (11349‐1‐AP, Proteintech), GDH (14299‐1‐AP; Proteintech). Secondary antibodies (Jackson ImmunoResearch Laboratories) were applied after rinsing for three times with PBS containing 0.1% Tween‐20. Blots were scanned by a Western Blot Imaging System (Sagecreation) and quantified by ImageJ (NIH).

### 4.9 Primary neuron and astrocyte coculture

Hippocampus of postnatal day 0 C57BL/6J pups were dissected under the microscope and cut into pieces. Then, the tissue was digested by trypsin bathed with 37°C water for 5 min and suspended. After 5 min centrifugation, 100 µL plating medium containing cells was plated on 12 mm coverslips coated with matrix gel (Corning) in 35 mm dishes. Two microliters of plating medium was added to the dishes 2 h later. A hundred milliliters of plating medium consist of 89 mL Minimal Essential Medium (Invitrogen), 10 mL fetal calf serum, 0.5 mM glutamine, 2 g NaHCO_3_, 0.5 g glucose, 10 mg bovine transferrin (Calbiochem), and 2.5 mg insulin. Forty‐eight hours after the plating, half of the medium was replaced by feeding medium for twice a week. A hundred microliters of feeding medium consist of 97 mL Minimal Essential Medium (Invitrogen), 2 mL B27 medium supplement (Invitrogen), 10 mg bovine transferrin, 0.5 mM glutamine, 0.5 g glucose, 3 mM cytosine‐p‐arabinofuranoside (Sigma–Aldrich), and 2 g NaHCO_3_.

### 4.10 Confocal imaging

Images were acquired with fluorescence laser scanning confocal microscopy (Zeiss LSM980) at room temperature. Mitochondrial colocalization was assessed utilizing the Plugin Colocalization Indices of ImageJ (NIH), while the PCC was employed to gauge the efficiency of mitochondrial targeting.

### 4.11 Virus preparation

The AAV vector plasmid pAAVDJ‐GFAP‐mtACR‐EGFP was constructed by replacing the CMV promotor with GFAP promotor and the N1 vector with pAAVDJ vector. pAAVDJ‐GFAP‐mtACR‐Mcherry was constructed by replacing the EGFP with Mcherry. pAAVDJ‐human Synapsin I (hSyn)‐mtACR‐EGFP and pAAVDJ‐hSyn‐mtACR‐Mcherry were constructed by replacing the GFAP promotor with hSyn promotor. The viruses for control groups (pAAVDJ‐GFAP‐4cox8‐EGFP, pAAVDJ‐GFAP‐4cox8‐Mcherry, pAAVDJ‐hSyn‐4cox8‐EGFP, pAAVDJ‐hSyn‐4cox8‐Mcherry) were constructed by replacing mtACR with 4cox8. pAAVDJ‐DIO‐mtACR‐Mcherry and pAAVDJ‐DIO‐4cox8‐Mcherry were constructed by inserting the sequence of mtACR‐Mcherry and 4cox8‐Mcherry into pAAVDJ‐DIO vector. pAAVDJ‐DIO‐mtACR‐EGFP and pAAVDJ‐DIO‐4cox8‐EGFP were constructed by replacing the sequence of Mcherry with EGFP. The fluorescent indicator of Cl^−^ (pAAVDJ‐GFAP‐clomeleon, pAAVDJ‐GFAP‐4cox8‐clomeleon, pAAVDJ‐hSyn‐clomeleon, and pAAVDJ‐hSyn‐4cox8‐clomeleon) were constructed by replacing the mtACR‐Mcherry with clomeleon or 4cox8‐clomeleon. pAAV‐DJ plasmid encoded the replication and virus capsid structure protein by AAV rep and cap genes and the pHelper plasmid contains the essential subset of adenovirus genes. The three plasmids were cotransfected into HEK 293t cells by transfection kit (Biodragon). Three days after the transfection, the virus was harvested and titrated by quantitative real‐time PCR.

### 4.12 Estimating the equilibrium potential of mitochondrial membrane

The equilibrium potential of the membrane is decided by the sum of equilibrium potential of ions, which could be calculated by Nernst equation, described as:

$$\;V_{\mathrm{eq}}=\;V_{\mathrm{in}}-\;V_{\mathrm{out}}=\;-\frac{RT}{zF}•\ln\frac{{\left[C\right]}_{\mathrm{in}}}{{\left[C\right]}_{\mathrm{out}}}$$

*R* represents the Gas constant, *T* represents the temperature, *z* represents the charge of ion (such as *z* = −1 for Cl^−^), *F* represents the Faraday's constant. According to this equation, the equilibrium potential is decided only by the concentration in and out the membrane under normal condition. Because of the ion permeability of GtACR1 described above and the majority of Cl^−^ in physiology condition, when mtACR is activated, the equilibrium potential of mitochondrial membrane should be close to the equilibrium potential of Cl^−^.

### 4.13 Surgery and stereotactic injection

Adult male mice were anesthetized by pentobarbital (80 mg/kg) and fixed with a stereotaxic apparatus (RWD Instruments). Five hundred nanoliters of virus was injected into the right SNC (AP = −3.16 mm, ML = −1.13 mm, DV = −4.4 mm) with an automated microsyringe pump (Drummond) fixed with a glass pipette (Drummond) pulled by a micropipette puller (Shutter Instruments, USA). After injection, the glass pipette was kept at a standstill for 5 min and then retracted. An optical fiber (core = 200 µm, numerical aperture [NA] = 0.37, length = 5 mm; RWD Instruments) was implanted into the same site of virus injection with a stereotactic cannula holder (RWD Instruments) and then fixed with dental cement (Yamahachi).

### 4.14 Fiber photometry

The virus of glutamate, GABA or Ca^2+^ indicator with AAVDJ‐GFAP‐mtACR‐Mcherry or AAVDJ‐GFAP‐4cox8‐Mcherry were injected into the right SNc of adult male mice as protocol described above. An optical fiber (core = 200 µm, NA = 0.50, length = 5 mm; Inper) was implanted at the same site of injection. A multi‐channel fiber photometry device (Inper) was used for the photometry. A 20 µW, 470 nm blue light was delivered to the optical fiber during baseline and then a 40 µW, 470 nm blue light was delivered to the optical fiber to activate mtACR. A 5‐min baseline and 25‐min recording were performed on freely moving mice. Data were collected and analyzed with Inper data process (Inper). The fluorescent change (Δ*F*/*F*) was calculated as (*F* − *F*_0_)/*F*_0_, where *F*_0_ is the baseline fluorescence signal. In order to eliminate the effects of baseline drift, the raw fluorescence trace was corrected as described by Xiao et al.^57^

### 4.15 Rotation test

Mice received virus injection and fiber implantation were monitored in an open fiend box. A 5‐min baseline was performed before photo‐stimulation. Then, a photo‐stimulation (533 nm, 25 mW/mm^2^, 1 min on, 1 min off) was performed on the mice. The rotation of each epoch was analyzed by EthoVision XT (Noldus). The normalized ratio of contralateral rotation was calculated by (*n*_contra_ — *n*_ipsi_)/(*n*_contra_ + *n*_ipsi_).

### 4.16 Drug delivery

GABA antagonist CGP35348 (HY‐103530; MCE) and glutamate antagonist CNQX (HY‐15066; MCE) were delivered by intraperitoneal injection 30 min before behavior test.

### 4.17 Adhesive remove test

Mice were habituated in a cylinder for 30 min 1 day prior to the test. Then, an adhesive strip was applied on the ipsilateral or contralateral forepaw of mice received virus injection. The time of mice removed the adhesive strip after feeling it was recorded.

### 4.18 Daily intermittent optogenetic stimulation test

Mice received virus injection and fiber implantation were subjected to a 10‐min baseline behavioral recording. Following this, the mice received 1 h/day photo‐stimulation (533 nm, 25 mW/mm^2^, 1 min on, 1 min off) for 2 weeks. A 10‐min behavioral test was conducted and recorded poststimulation.

### 4.19 Immunohistochemistry

Mouse was euthanized with an overdose of pentobarbital sodium and perfused with 0.9% saline and 4% paraformaldehyde. The brain was removed and incubated in 4% paraformaldehyde overnight at 4°C. Then, the brain was dehydrated with 30% sucrose and cut to 30‐µm thick sections by a freezing‐microtome (Leica CM 3050S). The sections were then incubated in 0.1% Triton X‐100 for permeabilization and blocked with 5% goat serum. Then, the slices were incubated with the primary antibodies against TH (25859‐1‐AP; Proteintech), GFAP (ab4674; Abcam), MAP2 (ab5392; Abcam), iba1 (ab178847; Abcam), α‐syn (ab51253; Abcam), and GABA (ab175; Abcam) overnight at 4°C. After washing for three times, the slices were incubated with the secondary antibody. Fluorescent images were acquired with a laser scanning confocal microscope (Zeiss LSM980) or a fluorescence microscope (Olympus IX83). The images were processed and analyzed with ImageJ (NIH).

### 4.20 Statistical analysis

The two‐tailed unpaired Student's *t*‐test was used for statistical analyses. Data were expressed as mean ± s.d. in main text and mean ± s.e.m. in figures. Comparisons of multiple groups were evaluated by one‐way ANOVA followed by Dunnett's or Tukey's test. Dunnett's test was used to compare every mean with a control mean, while Tukey's test was used to compare every pair. The statistical significance was accepted at *p* < 0.05, indicated by asterisks (ns, no significance; *p < 0.05; **p < 0.01; ***p < 0.001; ****p < 0.0001).

## AUTHOR CONTRIBUTIONS

*Conceptualization*: J. S. K. and R. Z. Y. *Software*: J. S. K., R. Z. Y., Z. W., Q. L., S. W., and S. M. L. *Formal analysis*: J. S. K., R. Z. Y., and S. M. L. *Methodology*: J. S. K., R. Z. Y., S. M. L., D. D. W., D. H. L., and X. Y. M. *Investigation*: R. Z. Y., S. M. L., D. D. W., D. H. L., and X. Y. M. *Visualization*: R. Z. Y., S. M. L., J. S. K., and X. G. *Funding acquisition*: J. S. K., P. P. L., and S. A. L. *Project administration*: J. S. K. *Supervision*: J. S. K., R. Z. Y, L. W., and Y. X. *Writing—original draft*: S. M. L., R. Z. Y., and J. S. K. *Writing—review and editing*: R. Z. Y. and J. S. K. All authors have read and approved the final manuscript.

## CONFLICT OF INTEREST STATEMENT

The authors declare no conflict of interest.

## ETHICS STATEMENT

All animal experiments have been approved by the Institutional Animal Care and Use Committee of Zhengzhou University (2024‐KY‐0375).

## Supporting information

Supporting Information

## Data Availability

All data generated or analyzed in this study are included in this published article and the supplementary information. The materials generated during the current study are available from the corresponding author on reasonable request. We did not generate the scRNA‐seq datasets, which were retrieved from the Gene Expression Omnibus (GEO) datasets (GSE157783 and GSE187012).
