# Supplementary material for: Neurotransmitter accumulation and Parkinson's disease‐like phenotype caused by anion channelrhodopsin opto‐controlled astrocytic mitochondrial depolarization in substantia nigra pars compacta
Source: MedComm (2020). 2024 May 15;5(6):e568. doi: 10.1002/mco2.568 (PMC11094672; doi:10.1002/mco2.568)
Supplement: Supplementary file 1 — Supporting Information [file MCO2-5-e568-s001.docx]

**Neurotransmitter accumulation and** **Parkinson’s disease-like phenotype caused by anion channelrhodopsin opto-controlled** **astrocytic mitochondrial depolarization in *substantia nigra pars compacta***

Sen-Miao Li^1,2,3^, Dian-Dian Wang^1,2,3^, Dan-Hua Liu^1,2,3^, Xiao-Yan Meng^1,2,3^, Zhizhong Wang^6^, Xitong Guo^7^, Qian Liu^8^, Pei-Pei Liu^1^, Shu-Ang Li^1^, Songwei Wang^6^, Run-Zhou Yang^1*^, Yuming Xu^2,4,5*^, Longde Wang^2,4,5*^, Jian-Sheng Kang^1,9*^

^1^ Clinical Systems Biology Laboratories, The First Affiliated Hospital of Zhengzhou University, Zhengzhou, 450052, China.

^2^ Department of Neurology, The First Affiliated Hospital of Zhengzhou University, Zhengzhou 450052, China.

^3^ The Academy of Medical Sciences, Zhengzhou University, Zhengzhou, 450052, China.

^4^ NHC Key Laboratory of Prevention and Treatment of Cerebrovascular Disease, Zhengzhou University, Zhengzhou, 450052, China.

^5^ Henan Key Laboratory of Cerebrovascular Diseases, Zhengzhou University, Zhengzhou, 450052, China.

^6^ College of Electrical and Information Engineering, Zhengzhou University, Zhengzhou, 450052, China

^7^ Zhengzhou University of Technology, Zhengzhou, 450052, China.

^8^ North China University of Water Resources and Electric Power, Zhengzhou, 450052, China.

^9^ Lead contact

^*^Correspondence: yangrunzhou@sibs.ac.cn (R.Z.Y.), xuyuming@zzu.edu.cn (Y.X.), longde_wang@yeah.net (L.W.), and kjs@zzu.edu.cn (J.S.K.)

Jian-Sheng Kang, Clinical Systems Biology Laboratories, The First Affiliated Hospital of Zhengzhou University, Zhengzhou, 450052, China. ORCID ID: 0000-0002-2603-9718.


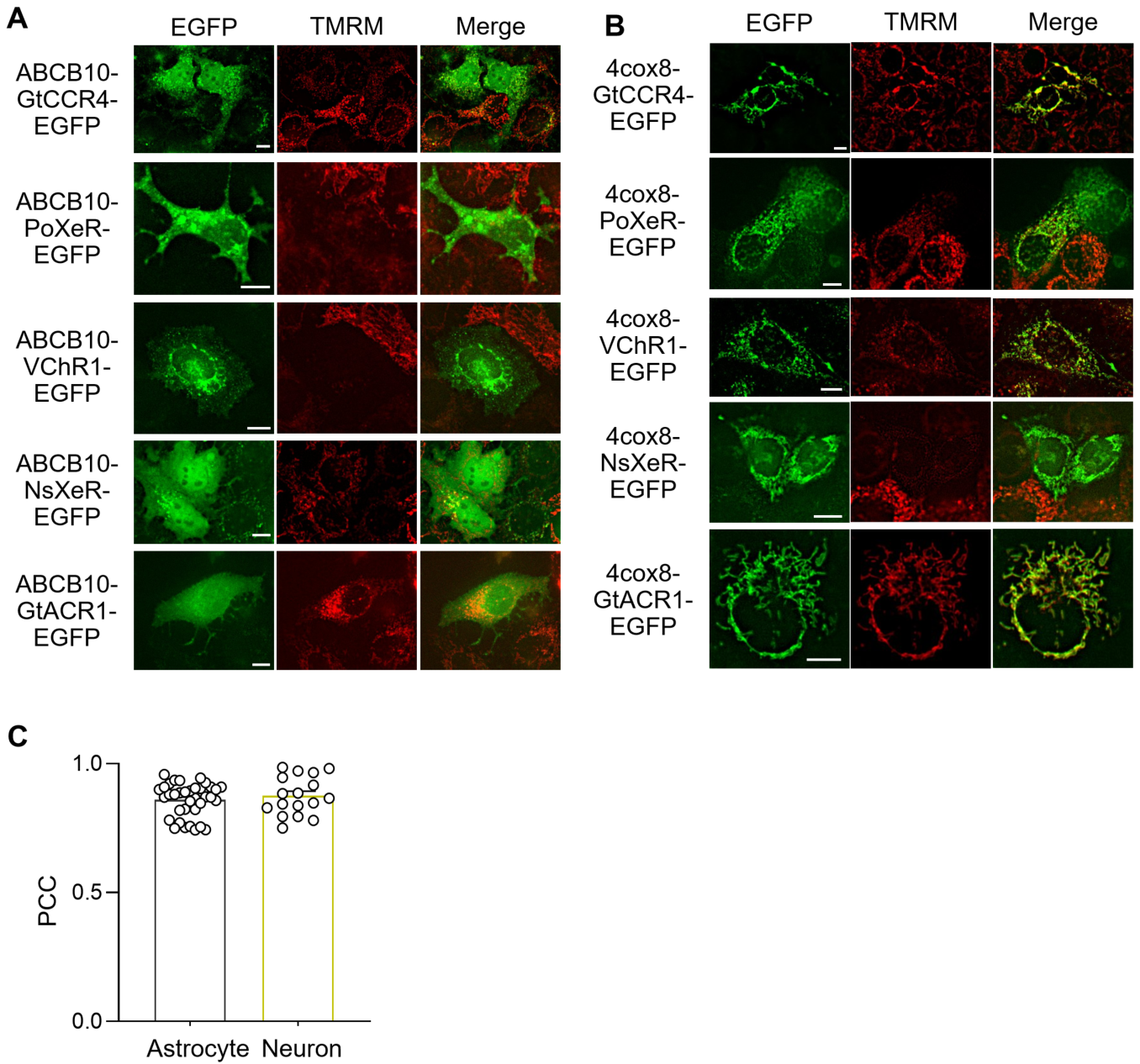


**Fig. S1. Mitochondrial targeting capabilities of various mitochondrial signal peptides fused channelrhodopsins.**

(A) Representative images of HeLa cells expressing fused channelrhodopsins, which were N-terminal fused with ABCB10 and C-terminal fused with EGFP. Scale bars, 10 μm. (B) Representative images of HeLa cells expressing fused channelrhodopsins, which were N-terminal fused with 4cox8 and C-terminal fused with EGFP. Scale bars, 10 μm. (C) Quantitative analysis of colocalization between mitochondria and mtACR in astrocytes and neurons using Pearson Correlation Coefficient (neuron, n = 17; astrocyte, n = 36).


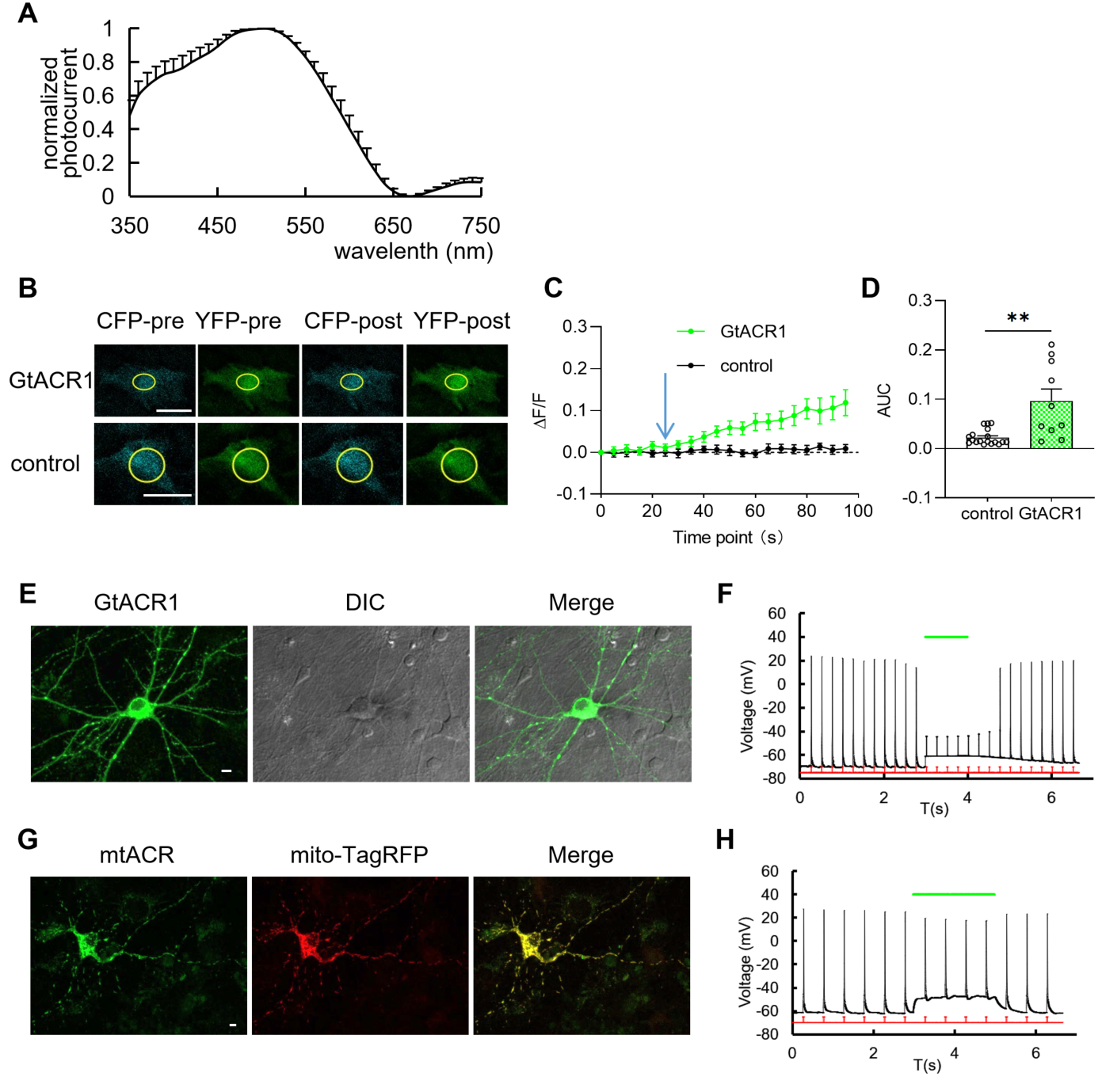


**Fig. S2. Electrophysiological properties of GtACR1.**

(A) Normalized photo-current responses of GtACR1 under photo-stimulations of various wavelengths. (B) Representative images of astrocytes co-expressing clomeleon with GtACR1 or only fluorescent protein. Scale bars, 10 μm. (C) Normalized fluorescent ratio of YFP/CFP changed under photo-stimulation. Black points represented control group (n = 18), while green points represented GtACR1 group (n = 10). (D) Area under the curves of fluorescent ratios of YFP/CFP. (E) Representative image of neuron expressing GtACR1. Scale bars, 10 μm. (F) GtACR1 under photo-stimulation inhibited action potentials of neuron. (G) Representative image of neuron expressing mtACR. Scale bars, 10 μm. (H) Action potential of neuron expressed mtACR remained normal under photo-stimulation. All data of figures in mean ± s.e.m.


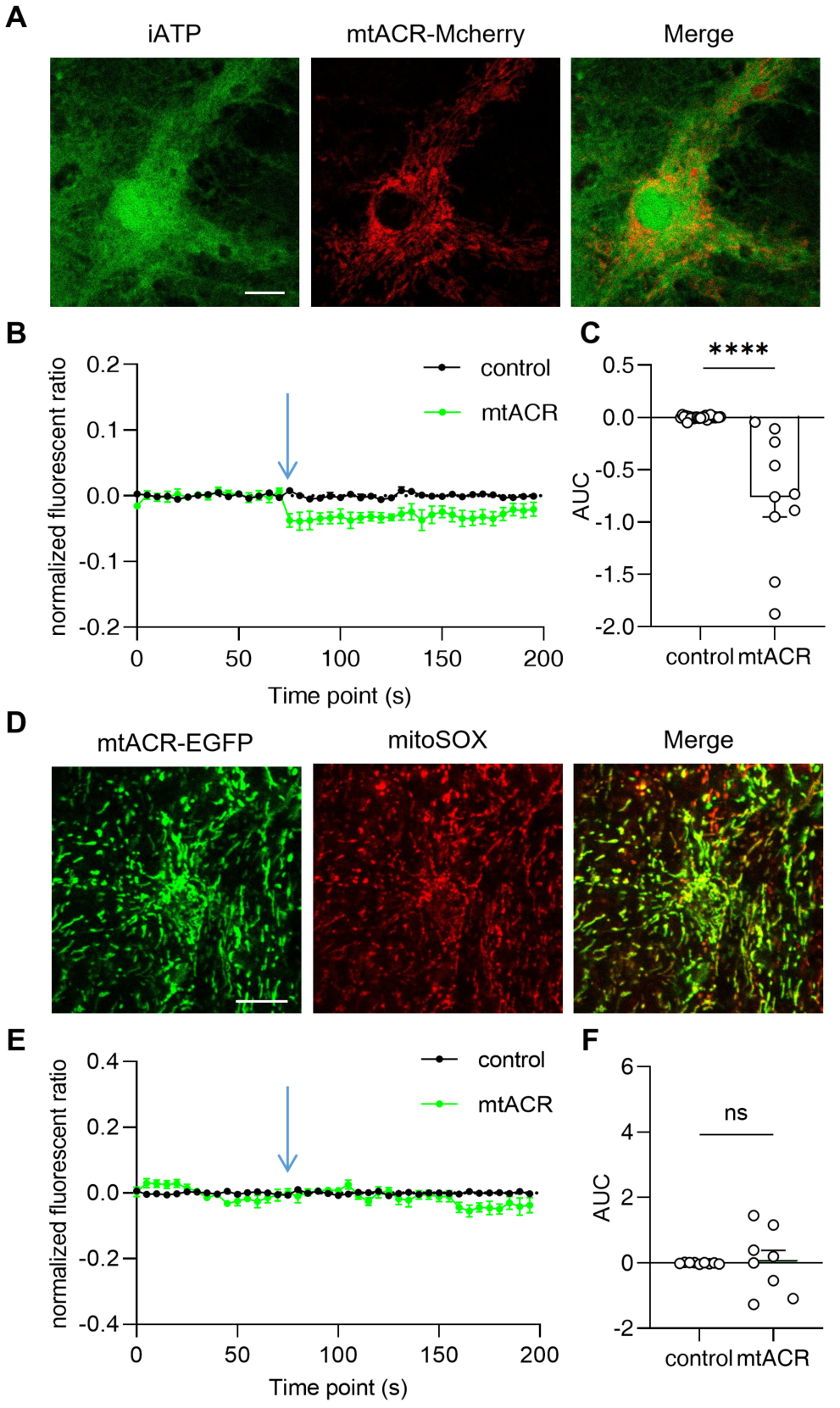


**Fig. S3. ATP and ROS dynamics following mitochondrial depolarization.**

(A) Representative images of astrocytes expressing iATP and mtACR-Mcherry. Scale bars, 10 μm. (B) Normalized ratio of iATP fluorescent intensity changed with photo-stimulation. Black represented control group (n = 25), while green points represented mtACR group (n = 10). (C) Area under curve (AUC) of fluorescent ratio of Rh800 (n = 25 for control group and n = 10 for mtACR group). (D) Representative images of astrocytes expressing mtACR-EGFP with mitoSOX staining. Scale bars, 10 μm. (E) Normalized ratio of mitoSOX fluorescent intensity changed with photo-stimulation. Black represented control group (n = 9), while green points represented mtACR group (n = 6). (F) AUC of fluorescent ratio of mitoSOX (n = 9 for control group and n = 6 for mtACR group).


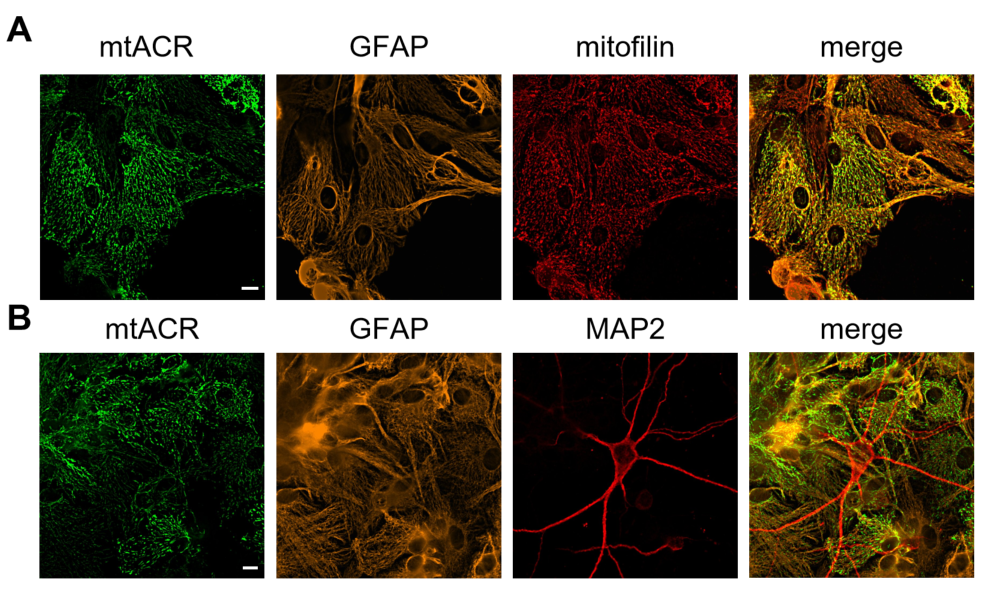


**Fig. S4. The expression of mtACR in astrocytes.**

(A,B) Representative images of GFAP and mitofilin (A) or GFAP and MAP2 (B) double-stained primary co-cultured neurons and astrocytes transduced with GFAP-mtACR-EGFP. Scale bars, 10 μm.


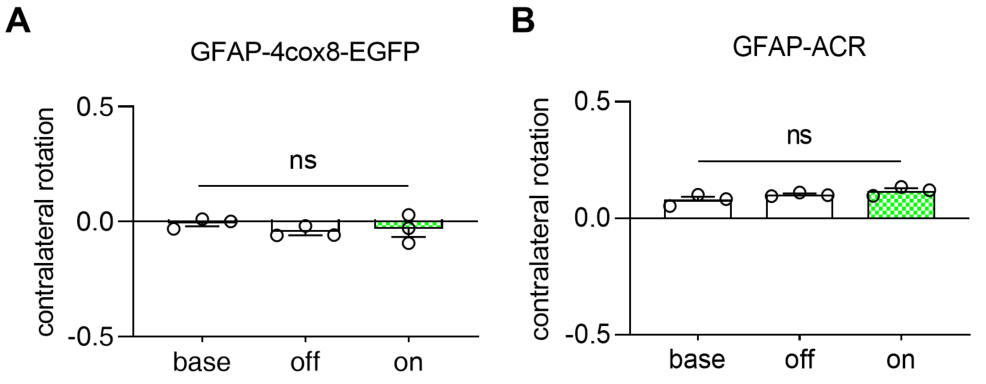


**Fig. S5. Behaviour tests of mice injected control virus.**

(A) Contralateral rotation quantifications of baseline, dark epochs, light epochs of mice injected control virus (GFAP-4cox8-EGFP) (n = 3). (B) Contralateral rotation quantifications of baseline, dark epochs, light epochs of mice injected ACR control virus (GFAP-ACR-EGFP) (n = 3). All data of figures in mean ± s.e.m.

**Fig. S6. Histopathological change of mice under transient photo-stimulation.**

(A, C, E) Representative images of GFAP (A), IBA1 (B) and TH (C) in SNc of mice under transient photo-stimulation. Scale bars, 100 μm. (B, D) Quantification of the number of astrocyte (B) or microglia (D) (n = 3). (F) Relative TH expression level in ipsilateral SNc compared to contralateral SNc of mice under transient photo-stimulation (n = 3). (G) Relative ratio of the DA neuron number in ipsilateral SNc compared to the contralateral SNc of mice under transient photo-stimulation (n = 3). All data of figures in mean ± s.e.m.


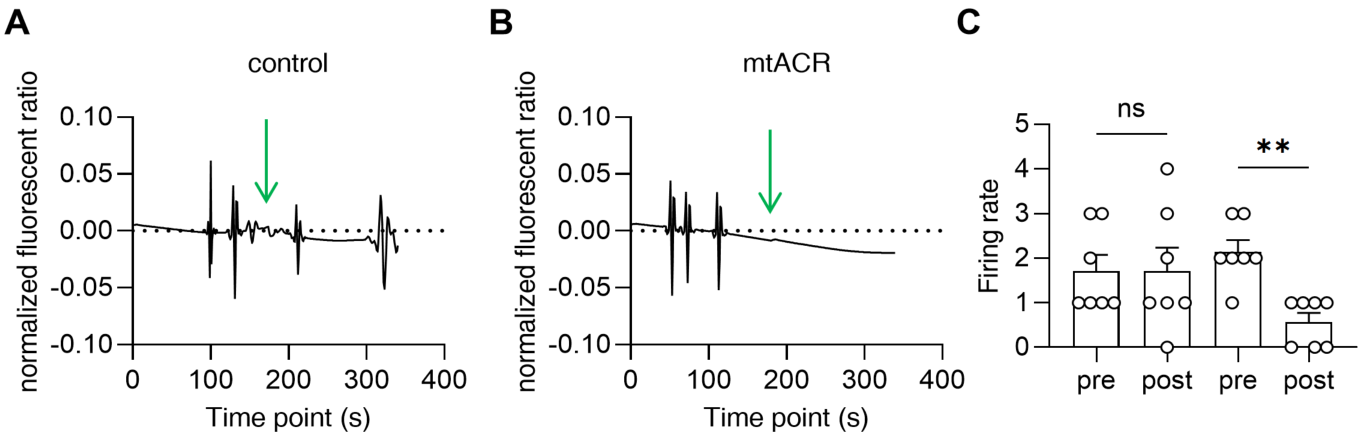


**Fig. S7. Firing rate of neurons following astrocytic mitochondrial depolarization.**

(A) Representative trace of fluorescent intensity of ASAP3 of mice received control virus injection. (B) Representative trace of fluorescent intensity of ASAP3 of mice received mtACR virus injection. (C) Quantitative analysis of neuronal firing rates (Data are mean ± s.e.m.; n = 3).


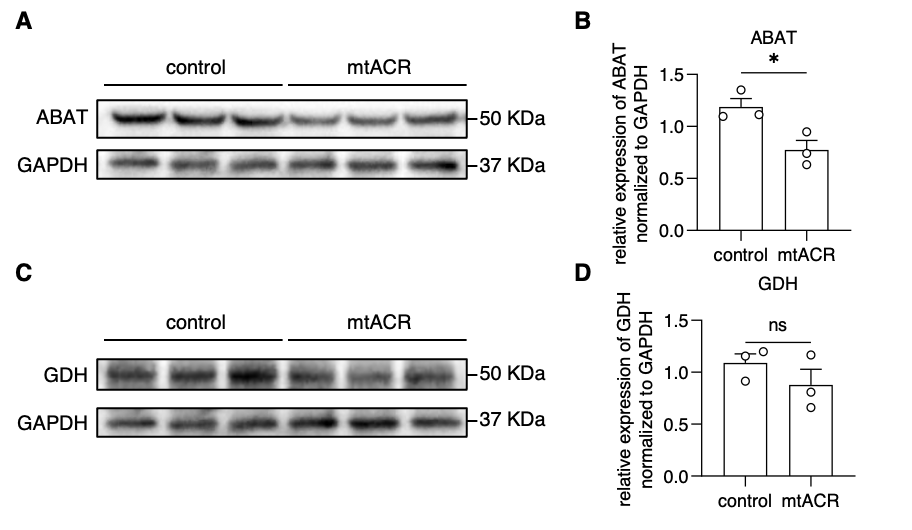


**Fig. S8. Change of enzymes following astrocytic mitochondrial depolarization.**

(A,C) Western blot of enzymes related to neurotransmitter metabolism.

(B,D) Quantitative analysis of the enzymes of mtACR group and control group (Data are mean ± s.e.m.; n=3).


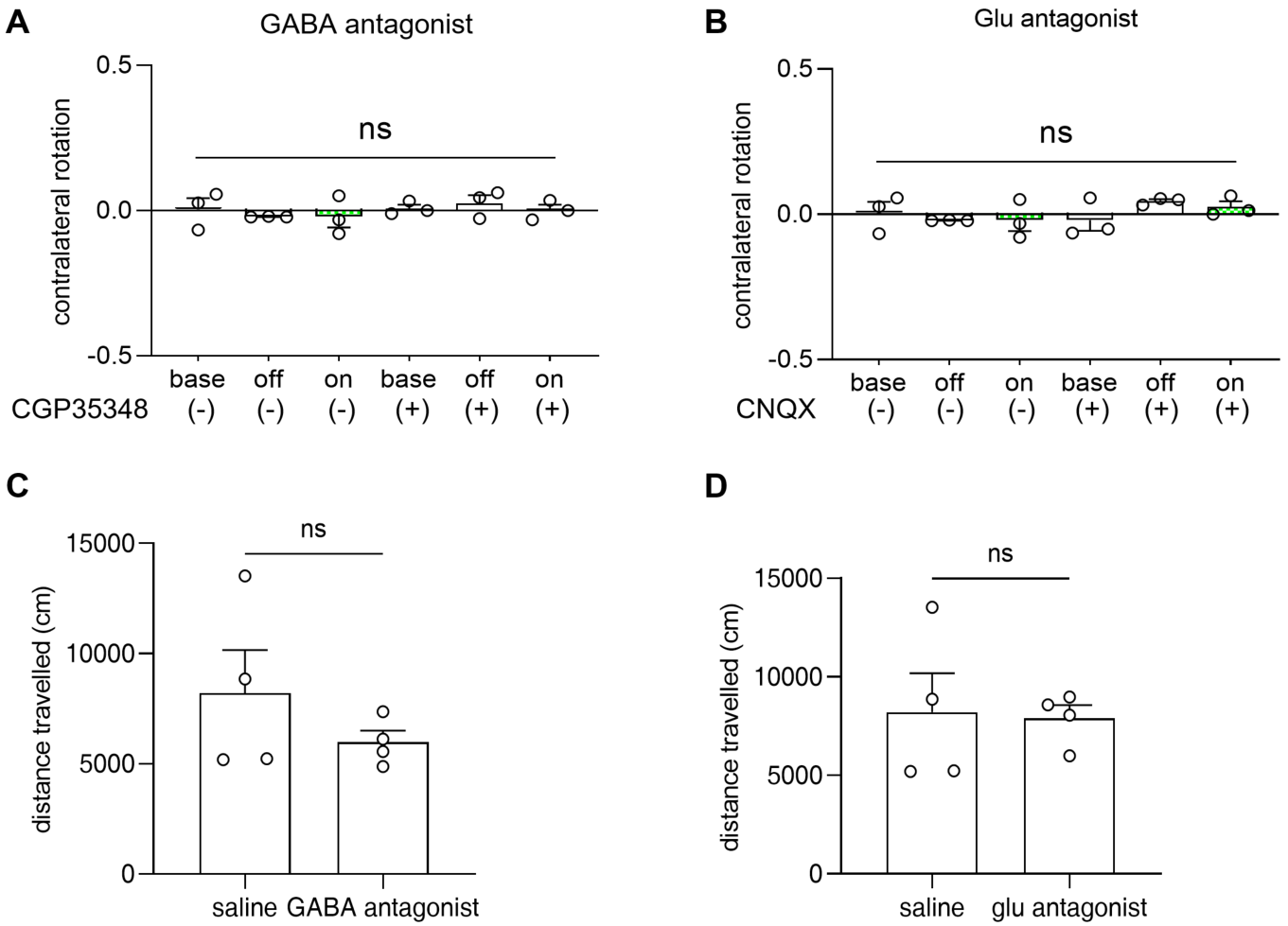


**Fig. S9. Behavior test of mice received neurotransmitter antagonists.**

(A,B) Contralateral rotation of mice injected control virus pre-treated with saline, CGP35348 (10 mg/kg) or CNQX (1.5 mg/kg) (i.p.) (Data are mean ± s.e.m.; n = 3). (C) Distance travelled of mice received saline or GABA antagonist CGP35348 (n = 4). (D) Distance travelled of mice received saline or glutamate antagonist CNQX (n = 4).


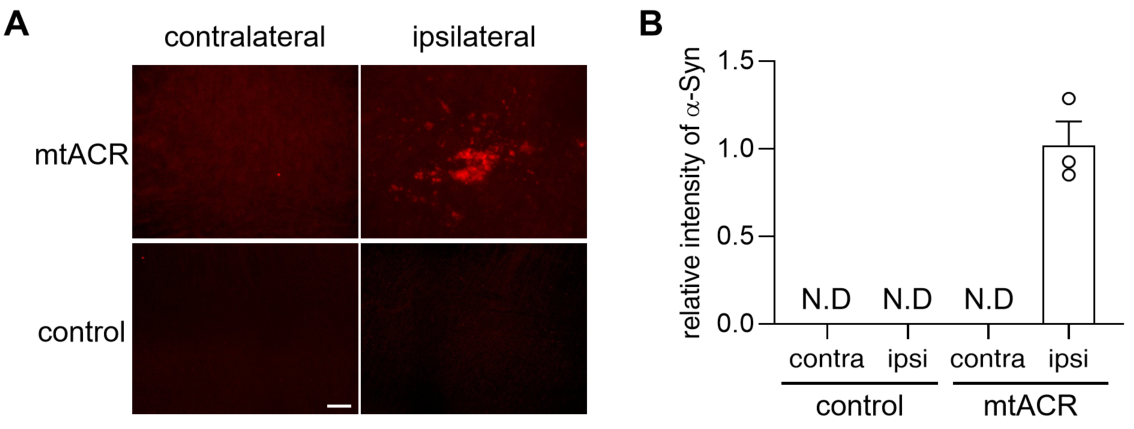


**Fig. S10. α-synuclein staining in SNc of mtACR mice or control mice.**

(A) Representative images of α-syn in the SNc of mice received mtACR or control virus injection. Scale bars, 100 μm. (B) Relative α-syn intensity in the ipsilateral and contralateral SNc of mice received mtACR or control virus injection (Data are mean ± s.e.m.; n = 3).


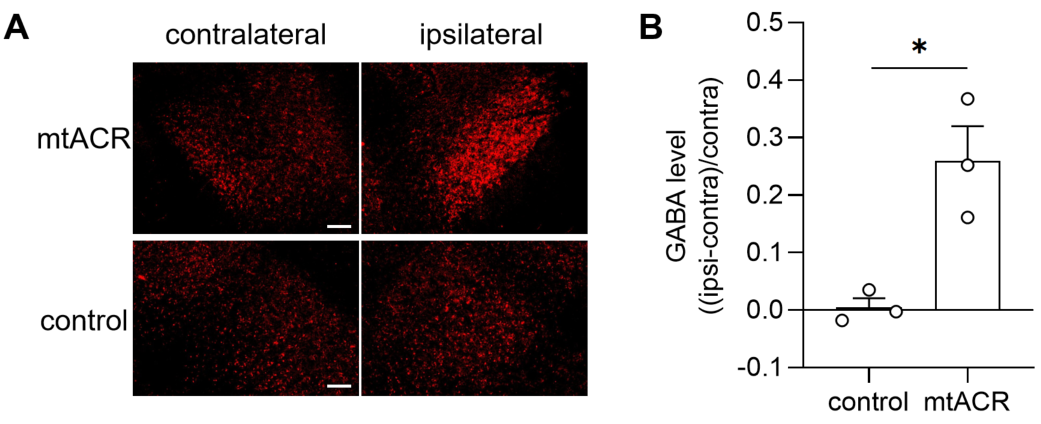


**Fig. S11. GABA staining in SNc of mtACR mice or control mice.**

(A) Representative images of GABA in the SNc of mtACR mice or control mice. Scale bars, 100 μm. (B) GABA level in ipsilateral SNc compared to contralateral SNc of mtACR mice or control mice (Data are mean ± s.e.m.; n = 3).


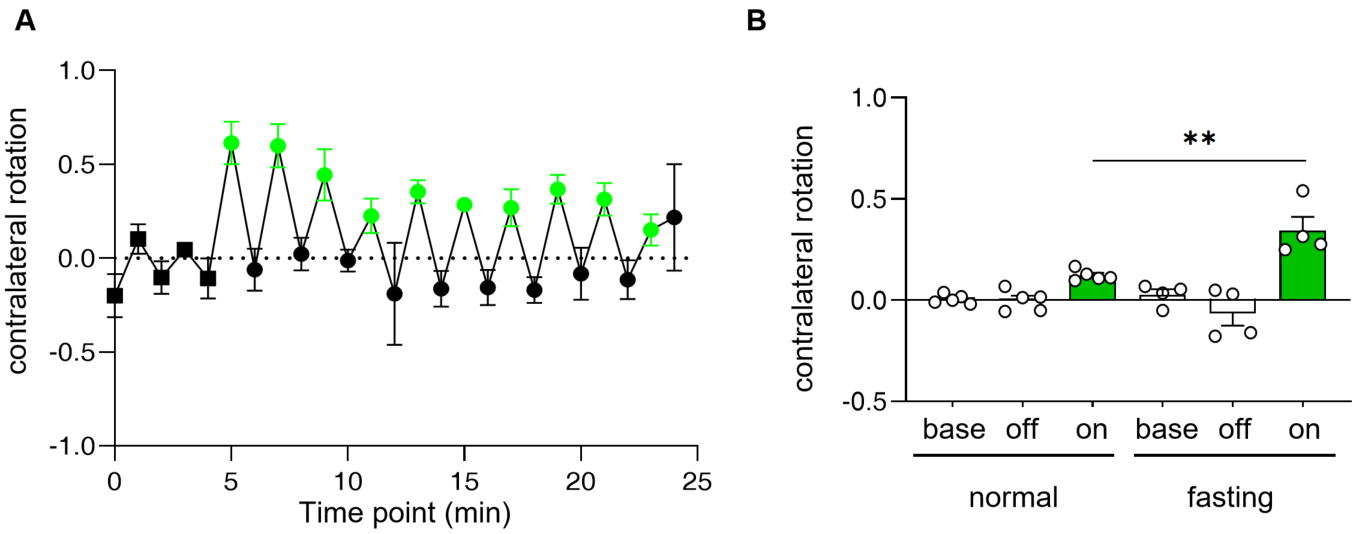


**Fig. S12. Astrocytic mitochondrial depolarization under mtACR photo-activation exacerbated locomotor deficit after mice fasted for 24 hours.**

(A) Contralateral rotation of each time point of behaviour tests of mice injected mtACR virus under photo-stimulation (n = 4). (B) Contralateral rotation of baseline, laser off epochs, laser on epochs of mice received mtACR virus injection (n = 4, **p < 0.01). All data of figures in mean ± s.e.m.

**Fig. S13. Mitochondrial related DEGs and enriched GO terms in astrocytes of PD patients.**

(A) Uniform manifold approximation and projection (UMAP) clustering of cells from the SNc of PD patients via single-cell RNA-seq. Clusters are labeled by each cell type. (B, C) Proportion of each cell type of control group (upper) and PD group (bottom). (D, E) Mitochondrial related DEGs and enriched GO terms of astrocytes.

**Fig. S14. Downregulated mitochondrial related DEGs and enriched GO terms in astrocytes of PD model mice.**

(A) Uniform manifold approximation and projection (UMAP) clustering of cells from the SNc of PD model mice via single-cell RNA-seq. Clusters are labeled by each cell type. (B) Downregulated mitochondrial related DEGs and enriched GO terms of astrocyte, microglia and neuron.

**Fig. S15. Upregulated mitochondrial related DEGs and enriched GO terms in astrocytes of PD model mice.**

Upregulated mitochondrial related DEGs and enriched GO terms of astrocyte, microglia and neuron.
